# Work Function Modulation of Molybdenum Disulfide Nanosheets by Introducing Systematic Lattice Strain

**DOI:** 10.1038/s41598-017-09916-5

**Published:** 2017-08-29

**Authors:** Jyoti Shakya, Sanjeev Kumar, D. Kanjilal, Tanuja Mohanty

**Affiliations:** 1 School of Physical Sciences, Jawaharlal Nehru University, New Delhi, 110067 India; 2 Inter University Accelerator Center, Aruna Asaf Ali Marg, New Delhi, 110067 India

## Abstract

Tuning the surface electronic properties of 2D transition metal dichalcogenides such as Molebdenum disulfide (MoS_2_) nanosheets is worth exploring for their potential applications in strain sensitive flexible electronic devices. Here in, the correlation between tensile strain developed in MoS_2_ nanosheets during swift heavy ion irradiation and corresponding modifications in their surface electronic properties is investigated. With prior structural characterization by transmission electron microscopy, chemically exfoliated MoS_2_ nanosheets were exposed to 100 MeV Ag ion irradiation at varying fluence for creation of controlled defects. The presence of defect induced systematic tensile strain was verified by Raman spectroscopy and X-ray Diffraction analysis. The effect of ion irradiation on in–plane mode is observed to be significantly higher than that on out-of-plane mode. The contribution of irradiation induced in-plane strain on modification of the surface electronic properties of nanosheets was analyzed by work function measurement using scanning Kelvin probe microscopy. The work function value is observed to be linearly proportional to tensile strain along the basal plane indicating a systematic shifting of Fermi surface with fluence towards the valence band.

## Introduction

Graphene derived materials have some remarkable properties with promising applications in electrical, mechanical, thermal as well as in optical devices^1, 2^. However, the absence of an intrinsic band gap in graphene hinders its applicability in electronic devices such as field effect transistor. On the other hand, layered transition metal di-chalcogenides (TMDs) are emerging as excellent alternative for the study of fundamental physics in two dimensional materials in layered crystals and uses in fabrication of optoelectronic devices. MoS_2_ is one of such kind of TMDs, which have drawn significant attention for their potential advantages in catalysis, transistor, photo detectors, gas sensors, batteries and other optoelectronic devices^3–8^. MoS_2_ is an indirect band gap (1.2 eV) semiconductor in bulk form^9^. The value of its band gap is layer dependent, which increases with decreasing number of layers and approaches to 1.8–1.9 eV for monolayer^9, 10^. In MoS_2_, the binding energy between adjacent planes is much lower than the binding energy within the plane and this fact has been exploited in various techniques such as chemical exfoliation, mechanical exfoliation, chemical vapor deposition etc that allows for the preparation of single and few layer MoS_2_ nanosheets^11–13^. Understanding of the surface potential and charge distributions on the surface of MoS_2_ is essential to enhance the performance of MoS_2_ based nano-devices. Tuning the work function (WF) or surface potential difference of 2D material has been reported to be a crucial step towards designing devices with desired performance^14^. Lattice strain has been observed to modify the WF of organic semiconductors^15^. Similarly, one can expect the modification of WF by introducing lattice strain in MoS_2_ nanosheets. Strain induced structural modification in bulk and nanomaterials using swift heavy ion (SHI) has been reported in literature^16^. SHI irradiation is one of the established methods to modify the optical, electrical and structural properties of nanostructured materials and nanocomposites thin films via defect creation^17–20^. The extent of property modification and damage fraction depend on mass, energy and charge state of the ions, its fluence and target density. Some of our earlier reported results on graphene based nanocomposites reveal shifting of Fermi level by SHI ion irradiation^19^. Similarly, one can introduce controlled defects in MoS_2_ nanosheets using SHI irradiation to tailor its optical and electronic properties. The defects so created can play a major role in inducing lattice strain in the system which on the other hand can affect the surface electronic properties. However, there is no experimental result reported till date regarding the tuning of work function of MoS_2_ nanosheets with lattice strain.

We, here, report for the first time the use of SHI irradiation in introducing controlled tensile strain in MoS_2_ nanosheets to modify its work function in a systematic manner. Tuning the WF of MoS_2_ has great potential for achieving optimized performance in flexible electronic devices. In this work MoS_2_ nanosheets have been synthesized using sono-chemical exfoliation method, which involves ultrasonication of MoS_2_ flakes in organic solvent (DMF), followed by centrifugation. These nanosheets were characterized initially by different spectroscopy techniques such as Transmission electron microscopy (TEM) and Field Emission Scanning Electron Microscopy (FESEM) for the analysis of surface morphology. To investigate the optical properties of the nanosheets Raman spectroscopy has been employed. Scanning Kelvin probe microscopy (SKPM) provided the WF measurement. SHI beams are expected to change the surface potential of the system. This change in the surface potential of the system significantly affects the surface electronic properties of the material. A distinct relation has been observed between lattice strain and electronic disorder in MoS_2_. This work will also be of immense importance for further theoretical study of strain related electronic properties of few layer TMDs where Density Functional Theory (DFT) calculation gets complicated as inter-layer van-der-Waal force has to be taken into account.

## Results and Discussion

**Figure 1:**
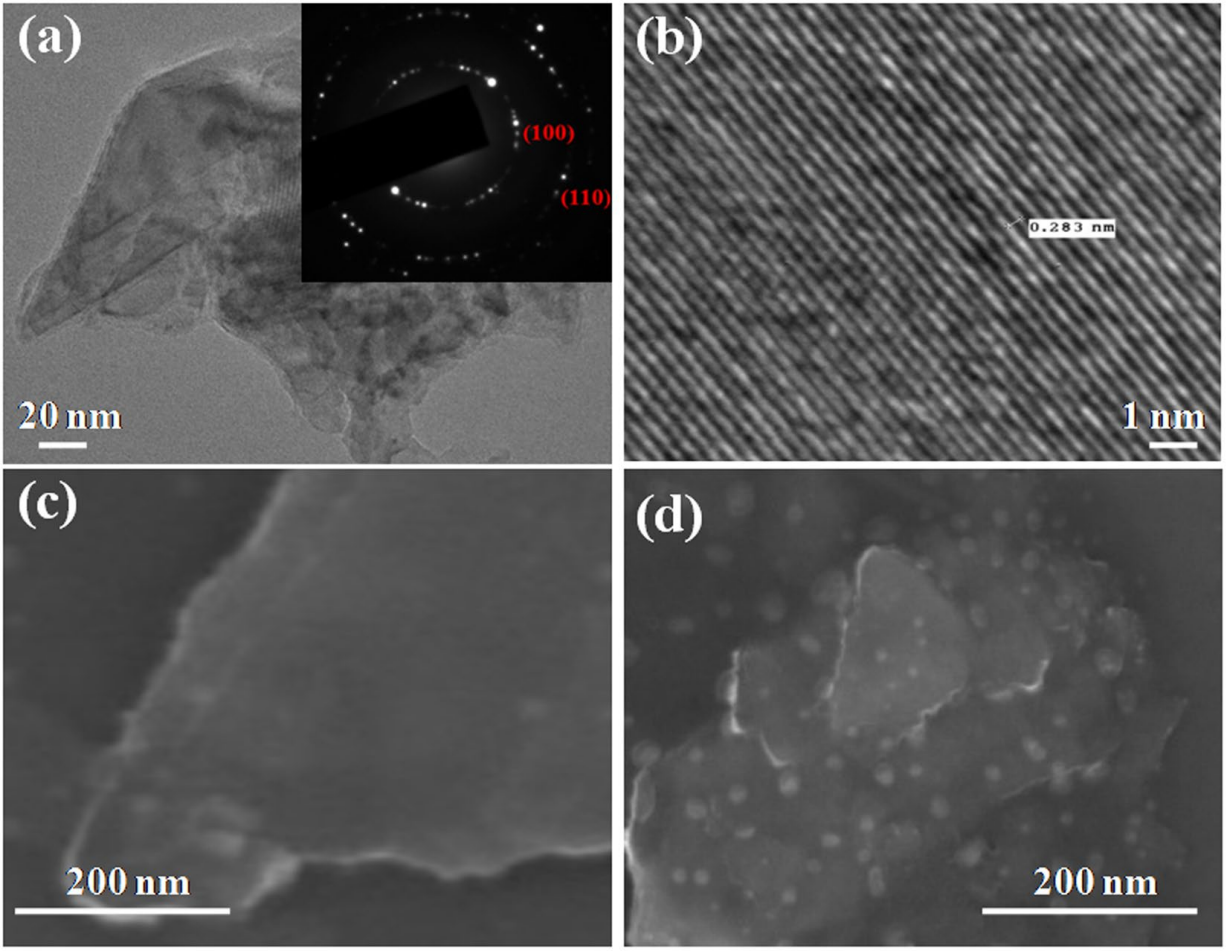
Morphological studies of pristine and irradiated MoS_2_. (**a**) TEM image of MoS_2_ nanosheets with inset showing selective area electron diffraction pattern (**b**) interplanar spacing in MoS_2_, (**c**,**d**) FESEM images of pristine and irradiated MoS_2_ nanosheets respectively.

Crystallinity of suspended MoS_2_ nanosheets (placed on TEM grid) was confirmed from TEM studies. TEM image of MoS_2_ nanosheets are presented in Fig. 1(a). Electron diffraction of nanosheets shows the atomic structure of planes present in MoS_2_ as shown in the inset of Fig. 1(a). High resolution TEM image showing the atomic structure of MoS_2_ is presented where a clear view of lattice fringes is shown in Fig. 1(b). The lattice spacing is of the order of 0.283 nm, which corresponds to (100) plane of MoS_2_^21^.

Layer morphology of pristine MoS_2_ is also confirmed from its FESEM image as shown in Fig. 1(c). Figure 1(d) exhibits surface morphology of MoS_2_ sheets irradiated at 5 × 10^13^ ions.cm^−2^. Formation of extended defects in MoS_2_ nanosheets in the form of latent tracks is clearly visible from FESEM image. Effect of irradiation induced disorders can be well reflected from modification in vibrational spectra of MoS_2_ nanosheets using Raman spectroscopy.

**Figure 2:**
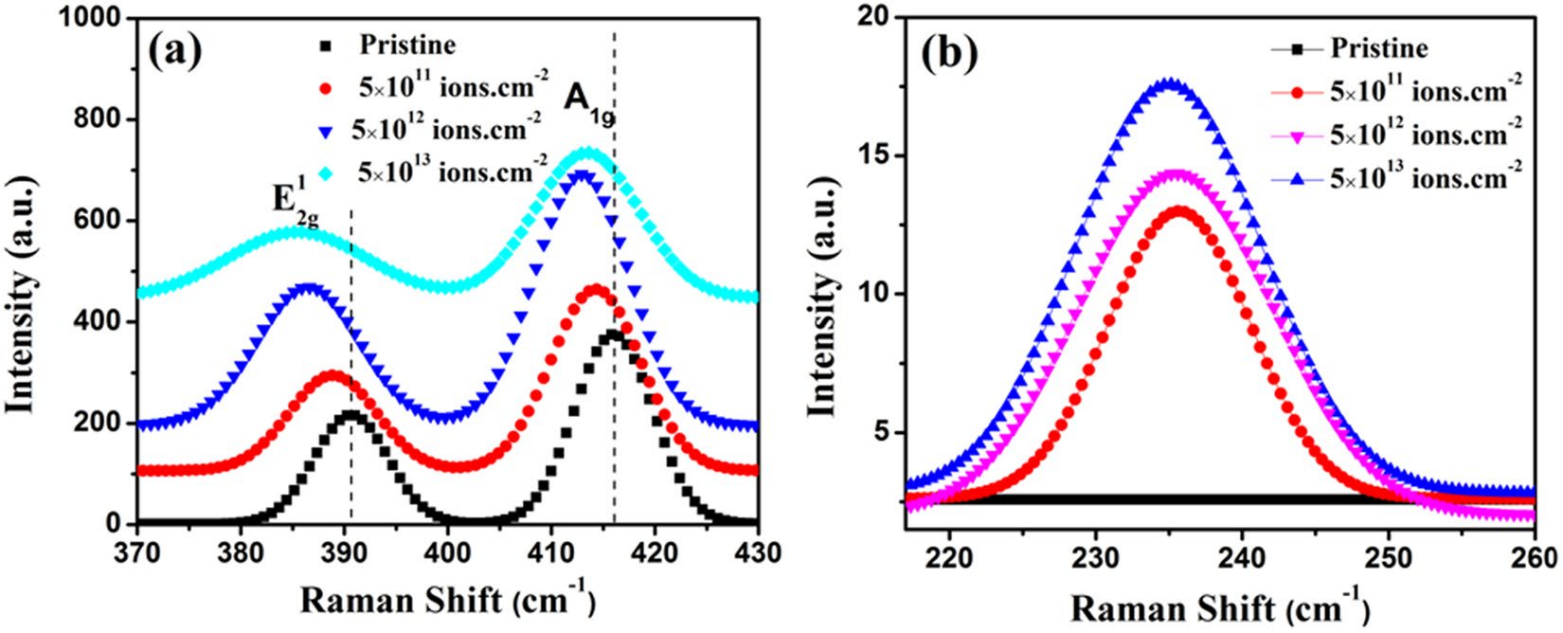
Raman modes of pristine and irradiated samples. Raman spectra of MoS_2_ nanosheets in (**a**) high frequency and (**b**) low frequency (showing defect induced LA peak) region.

Raman spectra of pristine as well as irradiated MoS_2_ nanosheets were recorded at five different spots covering almost full area using 532 nm laser excitation source with average power ~100 μW. Figure 2(a) shows Raman spectra of pristine and irradiated MoS_2_ nanosheets. The peak intensities have been obtained by fitting the peaks assuming Lorentzian distribution. Two prominent characteristic modes (E^1^_2g_ and A_1g_) are observed around 400 cm^−1^ (Fig. 2a). The in-plane E^1^_2g_ mode corresponds to opposite vibration of two S atoms with respect to Mo atom. Whereas A_1g_ mode arises due to the out of plane vibration of only S atoms in opposite direction^22^. The other modes like (E_1g_ and E^2^_2g_) of MoS_2_ could not be detected due to selection rule for scattering geometry (E_1g_)^1^ of the spectrometer or because of the limited rejection of the Rayleigh scattered radiation (E^2^_2g_)^2^ ^23^ However, in irradiated nanosheets, in addition to E^1^_2g_ and A_1g_ peaks, another Raman (LA) peak at 235 cm^−1^ corresponding to defects is also detected (Fig. 2b). The presence of this mode in irradiated MoS_2_ nanosheets corresponds to defects generated by SHI irradiation^24^. The sharp increase in peak intensity indicates the increase in number of defects with fluence. We have calculated the average inter defect distance ${{\rm{L}}}_{{\rm{D}}}=1/\sqrt{\sigma }$, where, σ is the density of ions impinging on the surface given by σ = it/Ae, here i is the ion current, ‘t’ is the exposure time to the ion beam, ‘A’ is the rastered area and ‘e’ is the elementary charge^24^. L_D_ decreases from 14 nm to 1.4 nm for fluence from 5 × 10^11^ to 5 × 10^13^ ions.cm^−2^.

The vacancies created per ion calculated Using SRIM 2008 Monte Carlo simulation for 100 MeV Ag ion on MoS_2_ target is found to be 106. [Parameters for SRIM calculation have been included in the Supplementary Information]. The values of induced defect density of the irradiated MoS_2_ under different ion beam fluences based on SRIM calculation are 5.52 × 10^16^ per cc, 5.52 × 10^17^ per cc and 5.52 × 10^18^ per cc corresponding to ion fluence 5 × 10^11^, 5 × 10^12^ and 5 × 10^13^ ions. cm^−2^ respectively.

**Figure 3:**
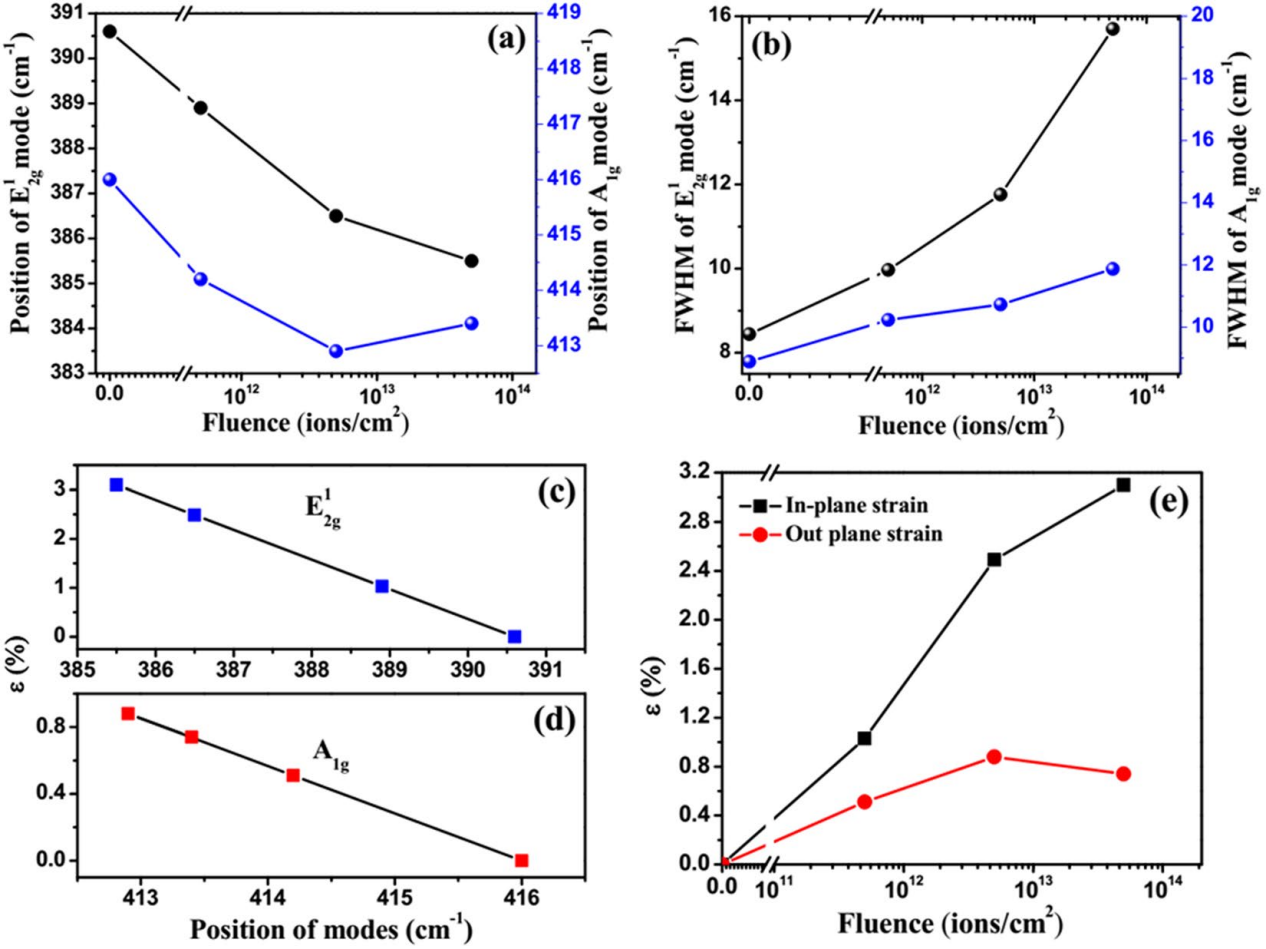
Investigation of Raman Spectra. (**a**) The change in position and (**b**) FWHM of ${E}_{2g}^{1}$ and A_1g_ mode with respect to fluence. (**c**) In-plane and (**d**) out of plane strain as a function of position of modes corresponding to different fluences, (**e**) Variation of strain with fluence.

Position of the E^1^_2g_ and A_1g_ peaks are plotted against the fluence in Fig. 3(a). In both the cases as compared to pristine MoS_2_ nanosheets, a systematic red shift for both ${E}_{2g}^{1}$ and A_1g_ modes were observed with increasing fluence and the magnitude of peak shift is more pronounced at higher fluences. This happens due to stretching of bond length which reduces the effective force between the atoms and hence the reduction of force constant between them leading to reduced frequency of vibration. This nature of peak shift rules out the possibility of charge transfer as the primary cause and point out the presence of tensile strain^24, 25^ leading to the increase in the bond length with fluence. The evolution of Raman spectra of MoS_2_ with strain is investigated from the variation of full width at half maximum (FWHM) with fluence as shown in Fig. 3(b). Lorentzian fitting of the peaks observed in Raman spectra provides information about the structural disorder via FWHM, thus indicating strain related variations. The broadening of two peaks was observed with increasing fluence which may be attributed to the lattice defects.

From the strain-induced shift of Raman-active phonon mode, we can measure strain using Raman Spectroscopy. Tensile strain can be expressed by the Gruneisen parameters (γ) which uniquely depends on the material. Gruneisen parameter is a remarkable physical quantity, which is related to the Raman shifts of atoms in a material in the microscopic field^21^. The strain can be calculated using the following formula^21, 26^.

1 $${\rm{\varepsilon }}=({{\rm{\omega }}}_{0}-{\rm{\omega }})/2{{\rm{\gamma }}{\rm{\omega }}}_{0}$$

where, ω_0_ and ω are the initial and shifted wave number respectively. We have taken γ $({E}_{2g}^{1})$ = 0.21 and γ (A_1g_) = 0.42^27, 28^. As previously discussed, both ${E}_{2g}^{1}$ and A_1g_ modes in MoS_2_ are very sensitive to fluence of ion beam irradiation. In our case, we observed maximum ${E}_{2g}^{1}$ peak shift of 5.1 cm^−1^ and A_1g_ peak shift of 3.06 cm^−1^ at a fluence of 5 × 10^13^ ions.cm^−2^ as compared to that of the pristine sample.

Figure 3c,d shows the variations in strain which is related to the change in the position of ${E}_{2g}^{1}$ and A_1g_ modes at different fluences in SHI irradiated MoS_2_ nanosheets. The position of both the peaks shifts linearly towards the lower wavenumber indicating the variation of tensile strain along a- as well as c- axis^29^. The in-plane mode under tensile strain gets stretched along a-axis and brings compression mostly along b-axis in the same plane which compensates the compression in the out-of-plane mode in the vertical plane because van der Waals force resists the compression, in the same way while it was under tensile strain^29^. The variation of strain in both A_1g_ and ${E}_{2g}^{1}$ mode as a function of fluence is plotted in Fig. 3(e). It is observed that, for A_1g_ mode the strain is increasing as a function of ion fluence until a maximum possible strain was attained and subsequently, it gets partially relaxed. It can be seen that the effect of ion irradiation on in–plane (${E}_{2g}^{1}$ mode) strain is significantly higher than that on out-of-plane (A_1g_ mode) strain. The difference in their magnitude increases with fluence. This difference can be attributed to the fact that in-plane (E^1^_2g_) mode involves vibration of both Mo as well as S atoms in the basal plane while in the out-of plane (A_1g_) mode only S atoms vibrate along c-axis and Mo atoms remains fixed, thus making A_1g_ mode stiffer as compared to in-plane mode. Our samples being few layers of stacked MoS_2_ nanosheets, one can expect the van der Waals interactions to produce more restoring force constant along c-axis causing gradual stiffening of out-of-plane mode and thus it (A_1g_ mode) gets less affected due to irradiation^30, 31^. This bond nature will affect the kind of defects induced in this system due to ion irradiation. It is reported that electron irradiation produces a number of defects (mostly vacancies) like V_s_, V_s2_, V_MoS3_, V_MoS6_ and S2_Mo_ etc. as maximum formation energy for them is 15 eV per atom in the basal plane of MoS_2_ nanosheets^32^. Therefore, it is quite obvious that 100 MeV Ag ion beam having high electron energy loss (S_e_) of the order of 17.26 keV/nm will create these kind of vacancies as well as extended defects in MoS_2_ in-plane mode resulting in expansion of in-plane Mo-S bond. In addition, normal incidence of ions on MoS_2_ nanosheets is expected to result in more number of defects in basal plane rather than along the out-of-plane direction of MoS_2_ nanosheets. Even then, high S_e_ value can generate a few number of defects along the out of plane mode which are reflected in increasing variation of A_1g_ mode with fluence which in due course gets relaxed beyond a fluence of 5 × 10^12^ ions.cm^−2^, where, the defects start overlapping (Fig. 3e).

**Figure 4:**
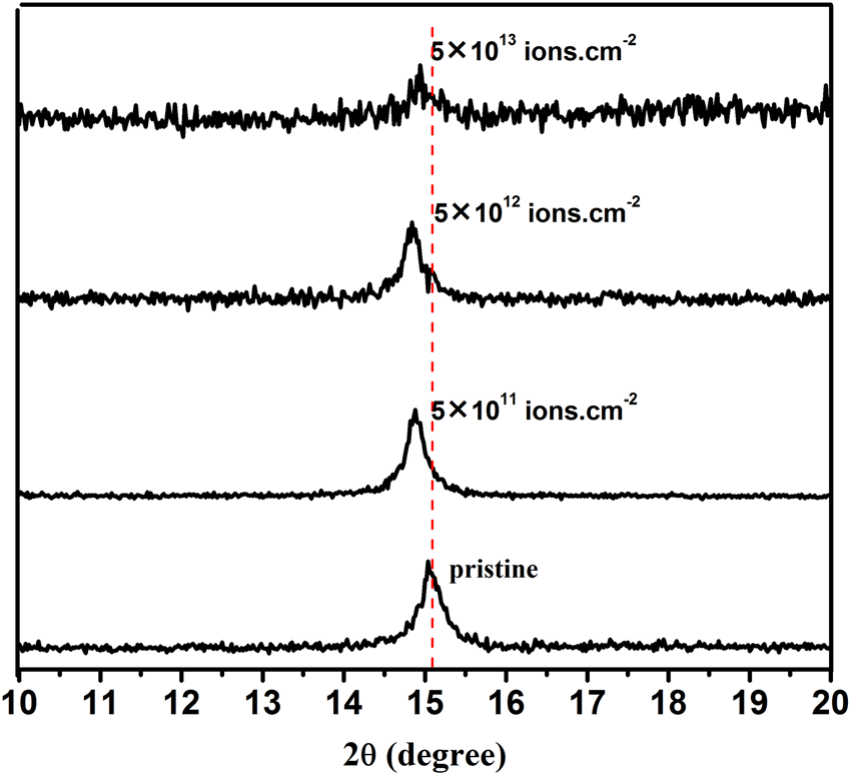
XRD spectra of pristine and SHI irradiated MoS_2_ for various fluences.

To reinforce the observed results in variation of strain with ion fluence, quantification of strain is also done using X-ray diffraction studies. XRD spectra of pristine and 100 MeV Ag ion irradiated MoS_2_ thin films at different fluences are shown in Fig. 4. The peak at 14.9° of (002) plane is the most prominent XRD peak of MoS_2_ nanosheets^33^. The changes observed in the X-ray diffraction spectra of SHI irradiated samples, are due to the disordering of the original crystalline structure of MoS_2_. This (002) peak (at 14.9°) gets significantly diminished with increase in fluence showing the occurrence of complete amorphization beyond a fluence of 5 × 10^13^ ions.cm^−2^.

The peak of (002) plane (c-axis) shifts to lower angle, indicating that there is an out of plane expansion, however small, in the irradiated MoS_2_ nanosheets. The atomic plane spacing in the MoS_2_ nanosheets can be calculated using Bragg law^34^:

2 $$2\mathrm{dsin}{\rm{\theta }}={\rm{n}}{\rm{\lambda }}$$

Here ‘d’ is the atomic plane spacing, θ is the Bragg angle, λ is the wavelength of incident X-ray and n is the diffraction order. The calculated ‘d’ value (0.598 nm) for (002) is larger for irradiated sample as compared to the pristine MoS_2_ (0.594 nm). The larger d value for (002) plane represents the presence of tensile strain along [001] direction in the irradiated sample^34^.

By recording 2θ shifts of MoS_2_ (002) peak as a function of fluence and calculating the corresponding d-spacing, the total elastic strain was calculated from the following relation between FWHM (B) and lattice strain (ε)^35^,

3 $${\rm{\varepsilon }}={\rm{B}}/4\,\tan \,{\rm{\theta }}$$

where, θ is Bragg’s diffraction angle. It is not possible to find the in-plane strain in MoS_2_ nanosheets, as there exists no peak corresponding to this plane in XRD except (002) plane (Fig. 4). Therefore, we have compared the results obtained from XRD and Raman measurements only for out of plane strain in MoS_2_ nanosheets induced due to SHI irradiation. The results from these two methods are strikingly very close to each other as presented in Table 1.

**Table 1:** Comparison of out-of-plane strain in pristine and irradiated MoS_2_ nanosheets obtained from Raman and XRD Techniques.

Fluence (ions.cm^−2^)	Out-of-plane Tensile strain using Raman Technique (%)	Out-of-plane Tensile strain using XRD Technique (%)
Pristine	0	0
5 × 10^11^	0.51	0.57
5 × 10^12^	0.88	0.94
5 × 10^13^	0.74	0.70

### Mechanism of SHI interaction

**Figure 5:**
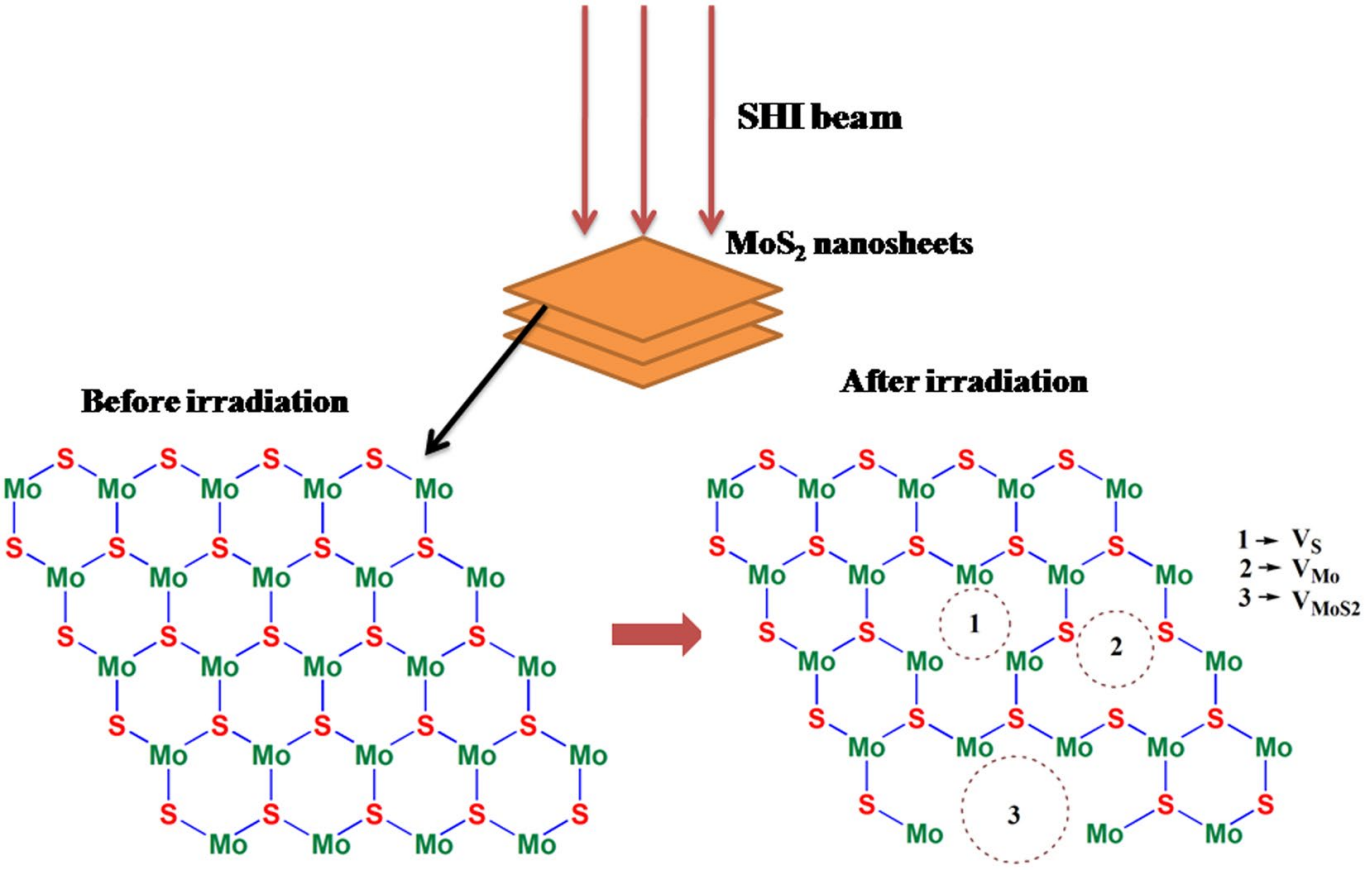
Schematic Top view of MoS_2_ before and after SHI irradiation showing creation of various vacancies.

The mechanism of defect creation on MoS_2_ nanosheets by SHI irradiation needs discussion. It is well reported that an energetic ion passing through matter loses its energy via nuclear energy loss (S_n_) and electronic energy loss (S_e_)^19^. Values of S_e_, S_n_ and range (R) of 100 MeV Ag ion irradiation on MoS_2_ nanosheets calculated using Monte Carlo simulations (SRIM 2008) were found to be 17.26 keV/nm, 0.101 keV/nm and 9.60 μm respectively. The ion interaction can result in creation of point defects, extended defects and even latent tracks in the target depending on above mentioned ion parameters. Variation of S_e_ and S_n_ with fluence is given in Supplementary information (Fig. S1). At keV energy range, predominant nuclear energy loss results in only point defects on the target. But in case of ions incident at MeV energy range, most part of the energy is lost through electronic excitation process. Normally two basic models such as Coulomb explosion and thermal spike model respectively are used to explain the early and later part of the ion interaction with matter^36^. On the basis of Coulomb explosion model relaxation of excess energy via radial impulses of atoms lying in the surroundings of ion path is expected to generate outward spreading of +ve ions away from the ion trajectory. These atomic movements give rise to radially expandable stress and strain around the ion track. Thermal spike model explains the relaxation of excess energy via energy transfer from excited electronic system to target atoms in a span of 10^−16^ s, thus leading to a local temperature increase followed by local thermalization via heat transfer from electron to lattice between 10^−14^ S to 10^−12^ s. Dense electronic excitation and efficient electron-phonon coupling can create a defected zone surrounding a ion trajectory termed as latent track. In the present situation, high value of S_e_ (17.26 keV/nm) is expected to create extended defects and even latent tracks in MoS_2_ nanosheets. From observed XRD results (Fig. 4) we could expect a complete amorphization of MoS_2_ nanosheets along the trajectory at a higher fluence beyond 5 × 10^13^ ions.cm^−2^. The amorphized region surrounding the ion trajectory is normally termed as latent track. Radius of these latent tracks can be estimated from the fluence dependent variation of any physical property of the nanosheets (Supplementary Information, Fig. S2). The schematic diagram of the effect of ion interaction on MoS_2_ is shown in Fig. 5. For convenience, only top view of the ion interaction with nanosheets is illustrated. SHI beams incident at normal angle on nanosheets are expected to generate defects, like vacancies, which are marked as circles in irradiated sample.

It is reported that SHI induced defects affect the work function (WF) of the oxide nanomaterials in a proportionate manner^19^. Similar effect can also be expected in SHI irradiated MoS_2_ nanosheets. The modification in WF of MoS_2_ nanosheets can be correlated to SHI induced strain in it. To further illustrate the WF modulation by strain, we investigated the variation of both tensile strain and WF with fluence. The variation of defect induced strain with fluence normally causes the Fermi level of the material to shift either towards valence band or conduction band^37^. The magnitude of shifting of Fermi level is estimated from the contact surface potential difference (CPD) measurement using SKPM.

**Figure 6:**
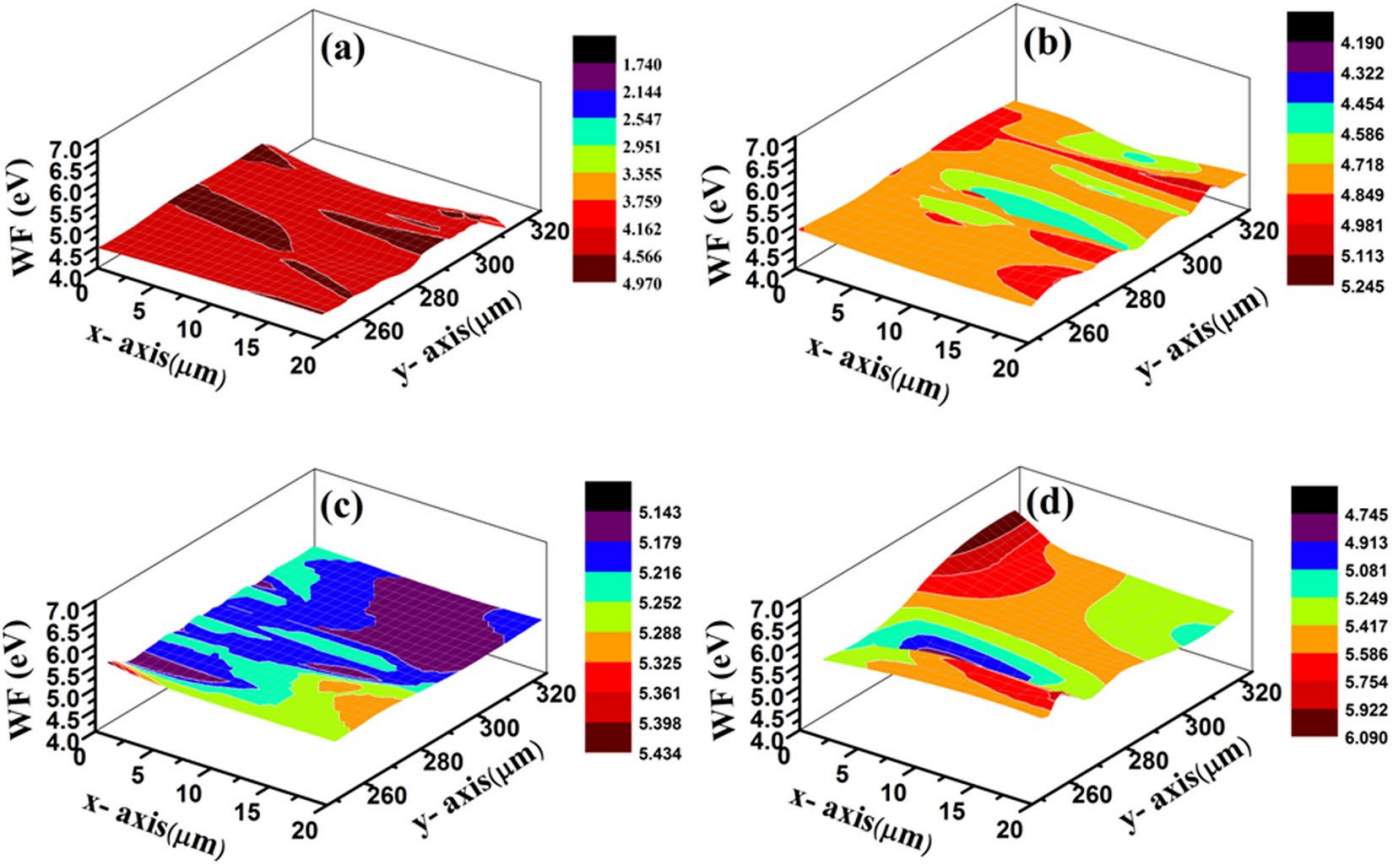
SKPM Analysis. Work function mapping of (**a**) pristine and irradiated MoS_2_ nanosheets by 100 MeV Ag ion beam at different fluences (**b**) 5 × 10^11^, (c) 5 × 10^12^ and (**d**) 5 × 10^13^ ions.cm^−2^.

The relation between CPD (V_CPD_) and WF can be expressed as^38, 39^

4 $${{\rm{eV}}}_{{\rm{CPD}}}={{\rm{\Phi }}}_{{\rm{tip}}}-{{\rm{\Phi }}}_{{\rm{sample}}}$$

where ɸ_tip_ is the known WF of reference Au tip and ɸ_sample_ is the WF of the sample. ‘e’ is the electric charge. The CPD mappings at different fluences are shown in the Fig. 6. Contour mapping is given in the supplementary information (Fig. S3). With increase in fluence, the CPD value decreases and corresponding WF increases. The measured CPD value for pristine MoS_2_ was found to be 561 mV. The CPD value for 100 MeV Ag ion irradiated thin film decreases from 313 mV to −381 mV for fluence varying from 5 × 10^11^ to 5 × 10^13^ ions.cm^−2^. Using equation 4, we find that the WF increases with increasing ion fluence for all irradiated MoS_2_ nanosheets. For pristine MoS_2_ it is calculated to be 4.53 eV. For increasing fluence in orders of magnitude (from 5 × 10^11^ ions.cm^−2^ onwards), the WF values are 4.78 eV, 5.21 eV and 5.48 eV respectively.

**Figure 7:**
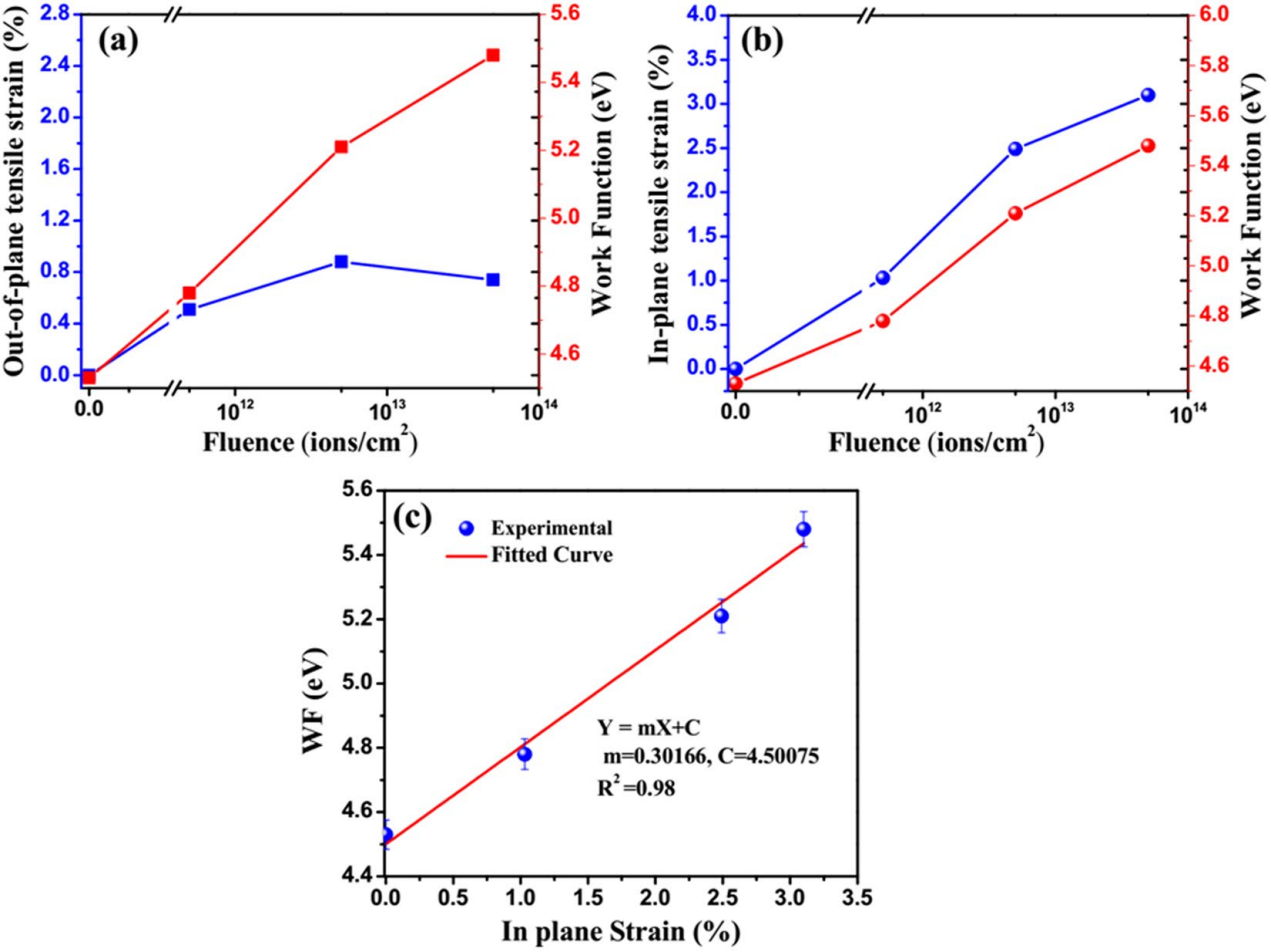
Relationship between WF and fluence dependent strain. Variation of (**a**) Out-of-plane and (**b**) In-plane tensile strain and WF with respect to fluence. (**c**) Linear dependence of the WF on in-plane strain.

To observe the correlation between lattice strain (in-plane and out-of-plane) and induced WF, all of them are plotted against the fluence. Figure 7a shows the variation of WF and out-of-plane strain with fluence. At low fluence up to 5 × 10^12^ ions.cm^−2^, both of them increases and beyond it there occurs a deviation in variation of out-of-plane strain with fluence having an indication of relaxation. The difference in variation could not establish any proportionate relation between WF and out-of-plane strain. The variation of work function and in-plain tensile strain with respect to the fluence is shown in Fig. 7b where both of them increase with fluence. Shifting of Fermi level towards the valance band is expected from the variation of WF with fluence.

Figure 7c gives the conclusive correlation between WF and in-plane strain of MoS_2_ where a linear relationship is found to be exist between them. This may be correlated to the roles played by defects generated in the wake of SHI irradiation. Due to the defect induced tensile strains, the separation between atoms gets increased and it causes the orbital overlap to lower down. When the atoms are moved farther apart from one another, the work function of the valence band maximum attains a more symmetrical shape and the location of the valance band maximum changes from K-point to the Г-point as the crystal is stretched^37^. This leads to decrease in valance bandwidth as well as Fermi energy level, which in turn increases the work function of MoS_2_ nanosheets.

In conclusion, introduction of controlled tensile strain using 100 MeV Ag ion beam for tailoring the surface electronic property of MoS_2_ nanosheets has been demonstrated. Raman and XRD results support the fact. The difference in variation of tensile strain along the in-plane and out-of-plane may be dependent on the nature of Mo-S bonds along a-b plane and c-axis. XRD results indicate the complete amorphization of the latent tracks in MoS_2_ nanosheets at higher fluence. The change in work functions of MoS_2_ as a result of induced tensile strain is estimated from contact potential difference measurements using scanning Kelvin probe microscopy. The work function of MoS_2_ nanosheets is found to be linearly dependent on the tensile strain along the basal plane. Our results suggest that the modification of the lattice strain by SHI irradiation provides an efficient way to engineer the band structure of MoS_2_. The results reported in this paper are relevant for further development of electronic applications of MoS_2_ as well as for device modeling. This work will also open up the way for DFT calculation for strain induced modification of work function of MoS_2_ nanosheets.

## Methods

Bulk MoS_2_ powder (Sigma-Aldrich) was dissolved in DMF (Sigma-Aldrich) solvent at a concentration of 0.5 mg/ml. The solution was sonicated for 6 h in ultrasonic bath. During the bath sonication process, there occur increase in pressure and temperature due to solvo-dynamic forces. These forces are capable of overcoming the van der Waals force between 2D layers, thus resulting in gradual exfoliation of layers from bulk MoS_2_. After sonication, the suspension was centrifuged at 1000 rpm for 15 min to obtain supernatant for further processing. The solution was drop casted on the silicon substrates. Subsequently, the samples were annealed at 153 °C for 1 hour to evaporate the solvent. The samples were then irradiated with 100 MeV Ag ions at different fluences (5 × 10^11^, 5 × 10^12^, 5 × 10^13^ ions.cm^−2^) using the 16 MV Pelletron accelerator at IUAC, New Delhi. Ion irradiations were carried out at normal incidence and room temperature. The pressure in the irradiation chamber was of the order of 10^−6^ torr. The average ion current was maintained at 2 particle nano ampere (1pnA = 6.25 × 10^9^ ions/s). The ion beam was magnetically scanned for uniform irradiation covering the complete sample surface. The range of the ion beam was ~9.6 micron which is much larger than the thickness of the nanosheets, thus avoiding the possibility of implantation of silver ions on nanosheets.

### Materials characterization

The pristine as well as SHI irradiated nanosheets were characterized by X-ray Diffraction (XRD), Field Emission Scanning Electron Microscopy (FESEM), Raman Spectroscopy and scanning Kelvin probe microscopy (SKPM) for structural, morphological, optical and surface electronic studies respectively. XRD measurement was done in a PANalytical X’pert pro set up with CuKα radiation (1.54 Å). FESEM images were taken using Tescan Model LYRA 3 XMU. Raman spectroscopic measurements were carried out by using WiTec Model alpha 300 having 532 nm excitation. The work function of pristine and SHI irradiated MoS_2_ were obtained using SKPM from KP Technology, United Kingdom. In addition, Transmission Electron Microscope (TEM, JEOL 2100 F) studies were carried out on suspended MoS_2_ nanosheets (on TEM grids) before they were deposited on the Si-substrate.

## Electronic supplementary material

Supplementary Information
