# Supplementary material for: Work Function Modulation of Molybdenum Disulfide Nanosheets by Introducing Systematic Lattice Strain
Source: Sci Rep. 2017 Aug 29;7:9576. doi: 10.1038/s41598-017-09916-5 (PMC5574977; doi:10.1038/s41598-017-09916-5)
Supplement: Supplementary file 1 — Supplementary Information [file 41598_2017_9916_MOESM1_ESM.pdf]

# SUPPLEMENTARY INFORMATION

## Work Function Modulation of Molybdenum Disulfide Nanosheets by Introducing Systematic Lattice Strain

Jyoti Shakya[1], Sanjeev Kumar[1], D. Kanjilal[2] and Tanuja Mohanty[1]*

[1]School of Physical Sciences, Jawaharlal Nehru University, New Delhi 110067, India

[2]Inter University Accelerator Center, Aruna Asaf Ali Marg, New Delhi 110067, India

*Correspondence and requests for materials should be addressed to Dr. T. Mohanty (Email: tanujajnu.com)

## Parameters for SRIM Calculations

The SRIM-2008 software package was used to calculate the projectile range, electronic energy loss ($S_e$) and nuclear energy loss ($S_n$) of ions and vacancies created per ion by using the following input data:

Parameters used are as follows:

Projectile Ion = Silver (Ag)

Mass = 106.905 amu, Energy = 100 MeV and Angle of incidence = $90^0$

Target = $MoS_2$

Density = 4.7811 g/cm$^3$ = 5.3960×10$^{22}$ atoms/cm$^3$

## SHI Interaction with Matter

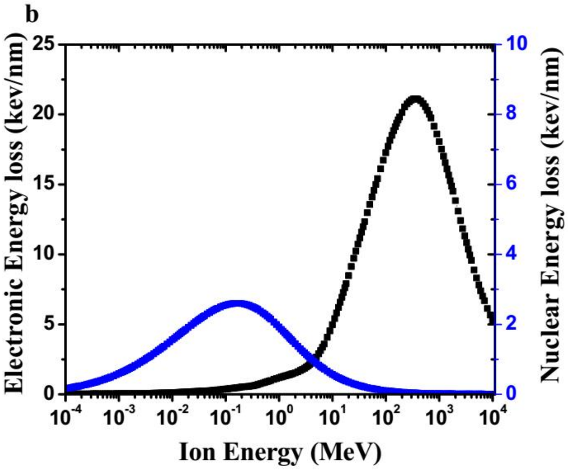


**Figure S1.** (b) Variation of Electronic and Nuclear energy loss with incident ion energy.

**Estimation of track radius:**

From XRD results we can expect a complete amorphization of the $MoS_2$ nanosheets in a surrounding volume along the ion trajectory. Assuming that latent tracks occupy the part of the specimen volume according to the Poisson law, the variation of work function with fluence can be expressed by equation $Y = Y_0 + A \exp(-X/B)$. Here, X is fluence, $Y_0 = 5.47$, $A = -0.87$, $B = 3.79\times10^{12}$

Thus, the damage cross section or area of a single ion track is $\sigma = 1/B = 0.26\times10^{-12}$ cm$^2$ where, latent track radius is found to be 2.82 nm.

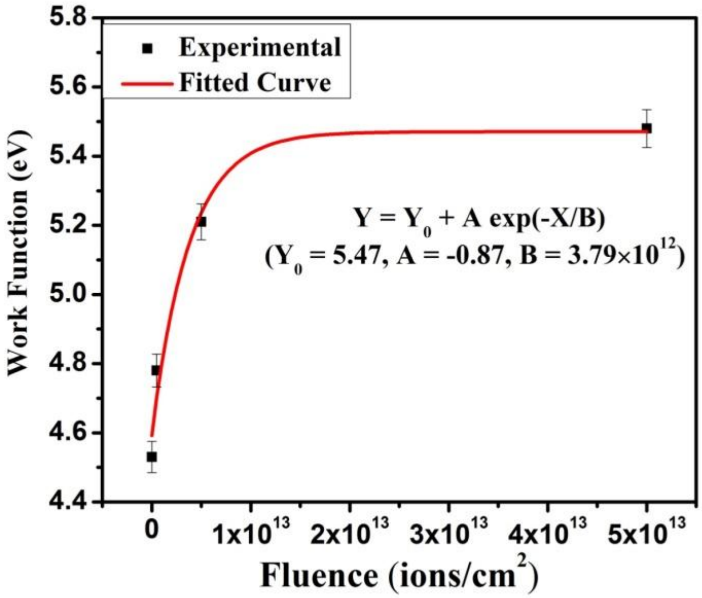


**Figure S2.** Variation of work function with Fluence.

**2D CPD mapping**

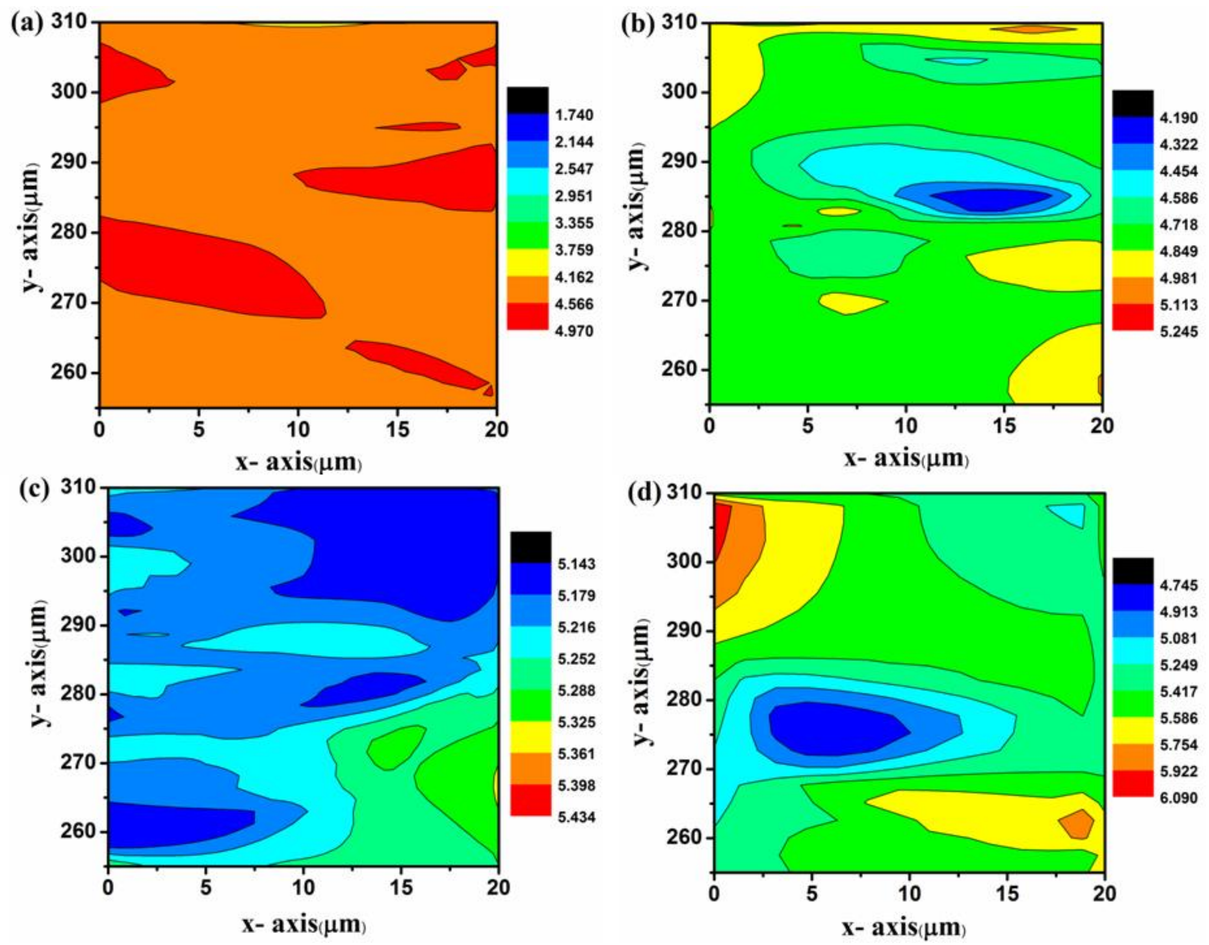


**Figure S3.** Variation of work function with Fluence (a) pristine, (b) $5\times10^{11}$, (b) $5\times10^{12}$ and (d) $5\times10^{13}$ ions. $cm^{-2}$
